# Role of laccase and xylanase, with or without ferulic acid esterase-producing *Lactiplantibacillus plantarum*, on the aerobic stability, microbial composition and in vitro degradability of mulberry silage

**DOI:** 10.1186/s12866-025-04165-3

**Published:** 2025-07-16

**Authors:** Ya Su, Qiang Yu, Yulong Xi, Yuanjiang Rong, Yixi Long, Yixiao Xie, Hong Sun, Jun Hao, Fuyu Yang, Yulong Zheng

**Affiliations:** 1 College of Animal Science, Guizhou University, 550025 Guizhou Guiyang, China; 2 Key Laboratory of Animal Genetics, Breeding & Reproduction in the Plateau Mountainous Region, Ministry of Education, Guizhou University, Guiyang, Guizhou, 550025 China

**Keywords:** Mulberry ensiling, Ferulic acid esterase, Lactic acid bacteria, Laccase and Xylanase, Aerobic stability, Bacterial community

## Abstract

Laccase (L), xylanase (X), and ferulic acid esterase (FAE) act on lignin - carbohydrate complexes. Whether these enzymes, alone or combined, can improve mulberry ensiling and aerobic stability is unclear. This study assessed the effects of L, X, and FAE - producing *L. plantarum* (LP) on whole - plant mulberry silage’s fermentation quality, aerobic stability, and microbial communities during aerobic exposure. After 60 days of ensiling, mulberry silage treated with CK, LP, LX, or M was unsealed for 1, 3, 5, or 7 days for exposure to air. The results indicated that the LP and M treatments decreased mulberry silage pH. The lower aminopeptidase and carboxypeptidase activities in the LP and M treatments might have contributed to the reduced degradation of crude protein (CP) and ammoniacal nitrogen (NH_3_-N) content (*P* < 0.05). Compared with the CK treatment, the addition of LX and M increased the acetic acid (AA) content by 1.49-2.68-fold, indicating greater aerobic stability (*P* < 0.05), which contributed to maintaining the storage quality of the silages during aerobic exposure. The application of additives to mulberry silage reduced the species richness; specifically, the additive treatments led to an increase in the relative abundance of *Kondoa* and *Lentilactobacillus* while decreasing that of *Enterococcus* and *Delftia*. Notably, *Lentilactobacillus* exhibited the capacity to inhibit the growth of other harmful microorganisms and emerged as the dominant genus within the LX group. In conclusion, treatment with the combination of laccase, xylanase, and FAE-producing *L. plantarum* can serve as an effective method to improve the silage quality and aerobic stability of mulberry.

## Introduction

China has long experienced a shortage of high - quality feed resources, especially high-quality protein feeds. This situation increases feed costs and reduces the efficiency of the livestock industry [1]. Therefore, developing new natural resources, such as nutrient - rich woody plants, is crucial for addressing the feed shortage resulting from increased animal husbandry. Mulberry (*Morus alba* L.) is rich in protein, minerals, bioactive substances, and other nutrients and can be considered a new resource for replacing traditional ruminant feeds and alleviating the shortage of traditional protein raw materials [2]. Moreover, it can improve animal health and the quality of products. For example, it can increase the amino acid and fatty acid content in meat, enhancing the nutritional value and market competitiveness of meat products [3].

However, mulberry silage lacks lactic acid bacteria (LABs), and has a high buffering capacity and lignocellulose level, leading to subpar ensiling results [4, 5]. Inoculation with LABs can improve fermentation, inhibit the growth of harmful microbes such as yeasts and moulds and increase the aerobic stability of silage [6]. The addition of cellulase and LABs can increase the quality of mulberry silage, conserve more nutrients and increase the antioxidant ability of the feed [7]. The LAB inoculation improved fermentation quality and aerobic stability of mulberry leaves silage, and the combination of *L. plantarum* and *P. pentosaceus* could serve as an effective LAB inoculant for improving the antioxidant capacity of mulberry silage [8, 9]. Using FAE-producing LABs in silage can promote lignocellulose decomposition and the release of free ferulic acid, enhancing silage quality and antioxidant properties [10]. Moreover, our team reported that the use of FAE-producing *L. plantarum* in combination with lignocellulose hydrolase significantly improved the fermentation quality of papyrus silage and decreases the lignocellulosic content in *Broussonetia papyrifera* [11].

Lignocellulose consists of lignin, cellulose, and hemicellulose linked by various chemical bonds, forming a complex matrix [12]. Lignin and hemicellulose increase the difficulty of cellulose degradation by forming lignin‒carbohydrate complexes around the cellulose [13]. Xylan, a hemicellulose, is located in the secondary wall and is the second most abundant plant polysaccharide [14]. Xylanase can degrade xylan into monosaccharides and sever lignin‒carbohydrate bonds [15]. Laccase is a copper-containing phenol oxidase that can oxidize lignin, form high-potential free radicals, and degrade lignin by opening the aromatic ring, resulting in an increase in reducing-sugar recovery during saccharification [16]. In silage, laccases can create anaerobic conditions for LAB growth, reducing storage losses from plant cell respiration and aerobic microbe metabolism [17]. FAE can break lignin‒hemicellulose linkages, increase the accessibility of degrading enzymes to lignocellulose, and improve the lignocellulose degradation rate and the FAE product can break the linkages between lignin and structural polysaccharides, thereby increasing the in vitro degradability of lignocellulose [18, 19]. The combined use of these three enzymes may increase the rate of degradation of the lignin-carbohydrate complex and promote the release of ferulic acid, which has strong antioxidant and antibacterial properties [20]. However, the combined use of these three enzymes in mulberry silage and their aerobic stability have not been reported. Therefore, whether the combination of FAE-producing *L. plantarum* with laccase and xylanase can have a positive effect on the aerobic stability of mulberry silage needs to be explored.

This study aimed to examine the synergistic effects of laccase, xylanase, and FAE-producing *L. plantarum* on mulberry silage by exploring the aerobic stability, protease activity, microbial composition, in vitro degradability, fermentation characteristics, antioxidant activity and microbial communities during aerobic exposure, thereby providing a basis for the preservation of mulberry.

## Materials and methods

### Raw materials and additives

The mulberry (second crop, tasseling stage) was harvested on 8 September 2023 at the experimental site in Qiantao Township, Huaxi District, Guiyang City, Guizhou Province. The experimental site, located at 106°43′- 106°50′ E and 26°17′- 26°24′ N, features a subtropical humid mesothermal climate, which is ideal for agriculture. It belongs to the subtropical humid and mild climate zone, with an average annual temperature of about 14–16℃. Its acidic yellow soil, with distinct physical and chemical properties, significantly impacts mulberry cultivation and yield. The FAE-producing *L. plantarum* used in this study were obtained from a previous isolation by our research team [21], the FAE activity of this strain is 163.82 nmol/min/ml. Xylanase (X, 100 U mg^−1^) was obtained from Shanghai Macklin Biochemical Technology Co., Ltd and laccase (L, 10 U mg^−1^) was obtained from Beijing Xiasheng Biotechnology Development Co., Ltd.

### Preparation of silage and aerobic stability analysis

Preparation of mulberry silage, the treatment groups were: Control group (CK) without the addition of bacteria and enzymes, Experimental group (LP) with the addition of FAE-producing strain *L. plantarum* at an addition of 1 × 10^6^CFU/g fresh matter (FM), the experimental group (LX) with the addition of 25 U/g FM laccase and 25 U/g FM xylanase, according to previous research [5, 22], and the experimental group (M) received supplementation with 25 U/g FM of laccase, 25 U/g FM of xylanase and 5 × 10^5^CFU/g FM of *L. plantarum*, respectively, and set up 4 replicates for each treatment. The bags were filled with 400 g/bag and then vacuum sealed for silage (store at room temperature away from light), the bags were opened after 60 days of silage and samples were taken at 0, 1, 3, 5 or 7 days after aerobic exposure to analyze the nutritional quality, fermentation quality and aerobic stability of each treatment, rumen in vitro fermentation after 60 days of ensiling.

After opening the bag, the feed is exposed to the air and covered with gauze to prevent contamination by other impurities such as fruit flies and moisture dissipation, and the gas can freely enter the silage bag. Multiple probes of the multi-channel temperature recorder (TOPRIE; Instrument Model: TP9000; Company: Shenzhen Toprie Electronics Co., Ltd.) are placed in the center of the feed, and three blanks are set at the same time, and the temperature is recorded at 30-minute intervals. If the sample temperature is higher than the ambient temperature by 2 °C, it means that the silage feed is beginning to rot and deteriorate, and the time is recorded [23].

### Fermentation parameters and chemical composition

The fermented mulberry silage samples were opened and mixed well, 10 g of each sample was weighed and put into a self-sealing bag, then 90 mL of sterile water was added, and leaching was carried out at 4℃ for 24 h, finally it was filtered through 4 layers of gauze to obtain the leachate of silage samples. It was stored in the refrigerator at −20℃ and then used to determine the pH value, organic acid and ammoniacal nitrogen content. The pH was determined using a pH meter (PHSJ-5T, INESA Scientific Instrument Co., Ltd., Shanghai, China). Organic acids, such as lactic acid (LA), acetic acid (AA), propionic acid (PA) and butyric acid (BA) were measured by liquid chromatography (Vanquish Core, Thermon Fisher Scientific, US) (KC-811 column; oven temperature: 50 °C; flow rate: 1 mL min^−1^) as the method of Li [24]. Ammoniacal nitrogen (NH₃-N) was determined using the phenol- sodium hypochlorite colorimetric method [25].

The 200 g of silage samples were taken and dried in an oven at 65℃ until constant weight to determine the dry matter (DM) content [26]. The dried samples were pulverized and sealed for storage. Crude protein (CP) was determined by the Kjeldahl method (VAPODEST500, C. Gerhardt GmbH & Co. KG, Germany) [26]. Neutral detergent fiber (NDF), acid detergent fiber (ADF), and acid lignin (ADL) were determined by the paradigm washed fiber method (ANKOM^DELTA^, ANKOM Technology, Macedon, NY, USA) [27]. Water-soluble carbohydrates (WSC) were determined by the enthrone- sulfate colorimetric method [28].

### Protein hydrolase activity

Upon the opening of the bag, 10 g of mulberry was sampled from each bag and homogenized with 90 mL of pre - cooled 0.1 M sodium phosphate buffer (pH 6.0, containing 5 mM sodium thiosulfate) for 60 s. The resulting homogenates were filtered through four layers of cheesecloth and subsequently centrifuged at 8,000 × g for 10 min at 4 °C. The supernatant samples were stored at − 80 °C for subsequent analysis of protease activities. The activities of aminopeptidase, carboxypeptidase, and acid protein - degrading enzyme were determined in accordance with the method described by Li [29].

### Analyses of microbial counts

The bacterial composition was analyzed using the plate counting method by mixing 20 g of sample with 180 mL of distilled water, shaking thoroughly, and performing serial dilutions (10⁻¹ to 10⁻⁷). Lactic acid bacteria were enumerated using De Man, Rogosa, and Sharpe (MRS) agar plates (Land Bridge, Beijing, China), which were incubated under anaerobic conditions at 37 °C for 48 h. Coliform bacteria were enumerated using Erythromycin Agar plates (Land Bridge, Beijing, China), which were incubated at 30 °C for 48 h. Yeasts were enumerated using Bengal Red Agar plates (Land Bridge, Beijing, China), which were incubated at 30 °C for 48 h.

### Microbial community analysis

Another 10 g of sample was divided into sealed bags and stored at −80 °C in a freezer for the identification of the microorganisms in the sample. To further elucidate the dynamic changes in microorganisms during silage fermentation, DNA was extracted from the samples using the standard cetyltrimethylammonium bromide (CTAB) method for sequencing analysis and strain identification. Polymerase chain reaction (PCR) amplification was conducted with specific primers, using Barcode and GC Buffer from New England Biolabs (Ipswich, MA, USA), as well as high-fidelity and high-efficiency enzymes. The bacterial 16 S rRNA gene was amplified using the primers F (5’-CCTAYGGGRBGCASCAG-3’) and R (5’-GGACTACNNGGGTATCTAAT-3’). The fungal ITS rRNA gene was amplified using the primers F (5’-CTTGGTCATTTAGAGGAAGTAA-3’) and R (5’-GCTGCGTTCTTCATCGATGC-3’). The bacterial 16 S amplification protocol was as follows: initial denaturation at 95 °C for 2 min, followed by 25 cycles of denaturation at 95 °C for 30 s, annealing at 55 °C for 30 s, and extension at 72 °C for 30 s, followed by a final extension was performed at 72 °C for 5 min. Small fragment libraries were constructed on the basis of the characteristics of the amplified regions; to determine the species composition of the samples, species annotation and abundance analysis of the validated data were performed by Novogene Technology Co., Ltd (Inner Mongolia, China). Subsequently, alpha diversity and coverage value analyses were conducted using the Magic platform (https://magic.novogene.com/) to assess differences in community structure.

### In *vitro* rumen fermentation trial

Rumen fluid was collected and filtered through four layers of gauze into preheated anaerobic thermos flasks and stored until use. In vitro digestion medium was prepared according to the methods of Zhou et al. [30]. Mulberry feed samples were reweighed [1.00 g dry matter] into polyester bags, sealed, and placed in 250 ml fermentation flasks. Two hundred milliliters of the rumen fluid mixture was subsequently dispensed into the preheated flasks at 39 °C, and the flasks were closed with plastic caps equipped with one-way valves to prevent the accumulation of fermentation gases after the flasks were filled with CO_2_. The flasks were then transferred to a shaker incubator and shaken at 125 r/min for 72 h at 39 °C. After 72 h of incubation, the nylon bags were removed and rinsed with water. The bags were then dried at 65 °C until a constant weight was reached. In vitro DM digestibility (IVDMD), NDF digestibility (IVNDFD), and ADF digestibility (IVADFD) were calculated from the change in weight after the reaction.

### Statistical analysis

Multifactorial analysis of variance (ANOVA) (one-way or two-way) was performed mainly on the fixed effects of additives and Aerobic exposure time using SPSS 21 software (IBM Corp., New York, NY, USA). Microbial data were normalized by log_10_-transformation on a fresh weight basis. Duncan’s and Tukey’s multiple comparison was used to determine the statistical difference between the means. Differences were considered significant when *P* < 0.05.

## Results

### Chemical composition of Raw material before ensiling

As shown in Table 1, before silage, the pH value of mulberry was 6.64, and the DM content reached 410.94 g/kg FM. The CP content was 115.43 g/kg DM, the NDF content was 409.24 g/kg DM, the ADF content was 275.98 g/kg DM, and the ADL content was 72.75 g/kg DM. Before ensiling, the aminopeptidase activity in mulberry was 17.32 units h⁻¹ g⁻¹ DM of forage, the acidic protein hydrolase activity was 69.03 units h⁻¹ g⁻¹ DM of forage, and the carboxypeptidase activity was 6.95 µmol h⁻¹ g⁻¹ DM. The mulberry had a WSC content of 111.97 g/kg DM and LAB counts of 5.90 log_10_ CFU g^−1^ FM, which met the ensiling requirements.

**Table 1:** Chemical composition and protein hydrolase activity of ensiling materials

Parameter	Mean
pH	6.64
DM (g/kg FM)	410.94
WSC (g/kg DM)	111.97
CP (g/kg DM)	115.43
NDF (g/kg DM)	409.24
ADF (g/kg DM)	275.98
ADL (g/kg DM)	72.75
Aminopeptidase (units h^−1^ g^−1^ DM of forage)	17.32
Carboxypeptidase (µmol of free amino acid released h^−1^ g^−1^ DM of forage)	6.95
Acid proteinase (units h^−1^ g^−1^ DM of forage)	69.03
LAB(log_10_ CFU/g FM)	5.90
*Coliform* bacteria (log_10_ CFU/g FM)	6.61
Yeast (log_10_ CFU/g FM)	4.70

*DM* dry matter, *FM* fresh matter, *WSC* water-soluble carbohydrates, *CP* crude protein, *NDF* neutral detergent fibers, *ADF* acid detergent fiber, *ADL* acid detergent lignin, *LAB* lactic acid bacteria, *CFU* colony-forming units

### Fermentation of silage during aerobic exposure

The data in Table 2 indicate that the addition of laccase, xylanase, and L. plantarum (LP, LX and M treatments) significantly reduced the pH value of the mulberry silage (*P* < 0.05). During the first 5 days of aerobic exposure, the pH values of the groups treated with FAE-producing *L. plantarum* (LP and M) were significantly lower than those of the groups without *L. plantarum* (CK and LX) (*P* < 0.05). During aerobic exposure, the LA and AA contents in all the additive treatment groups were consistently significantly greater than those in the control group (*P* < 0.05). The LA content increased by 6.46–44.09 g/kg DM, and the AA content increased by 0.46–1.13 g/kg DM. In particular, compared with the CK treatment, the laccase and xylanase treatments (LX and M) increased the AA content of mulberry silage by 1.49–2.68-fold. Additive-treated PA and BA levels were unsatisfactory, possibly due to temperature changes in the air during aerobic exposure. The effect of additive treatment on the contents of PA and BA is not ideal, possibly because the contents are lower than the detection values. The PA content in the M treatment increased on the 7th day of aerobic exposure, possibly due to the increase in pH value. The NH_3_-N content increased with the increasing aerobic exposure time, the treatment groups containing FAE-producing *L. plantarum* (LP and M) was significantly lower than that of the treatment groups without the addition of FAE-producing *L. plantarum* (LX and CK) (*P* < 0.05). The NH_3_-N levels were significantly lower in the LX treatment than in the CK treatment for the first 3 days of aerobic exposure (*P* < 0.05).

**Table 2:** Changes in fermentation during aerobic mulberry exposure

Parameter	Treatment (T)	Days of aerobic exposure (D)					SEM	*P*-value		
		Day 0	Day 1	Day 3	Day 5	Day 7		T	D	T × D
pH	CK	4.71^Ac^	4.83^Abc^	4.94^Abc^	5.76^Aab^	6.09^Aa^	0.087	<0.001	<0.001	0.001
	LP	4.20^Bb^	4.21^Cb^	4.26^Cb^	4.27^Bb^	4.97^Ba^				
	LX	4.66^Ac^	4.68^Bbc^	4.73^Bab^	4.76^Bab^	4.74^Ba^				
	M	4.17^Bb^	4.19^Cb^	4.21^Cb^	4.20^Bb^	5.53^ABa^				
LA (g/kg DM)	CK	40.34^Db^	54.36^Ba^	29.36^Dc^	13.24^Cd^	7.12^Ce^	0.380	<0.001	<0.001	<0.001
	LP	69.75^Bb^	89.35^Aa^	56.00^Bc^	25.87^Bd^	22.92^Ad^				
	LX	43.49^Cb^	59.05^Ba^	37.81^Cc^	23.41^Bd^	23.06^Ad^				
	M	83.43^Aa^	91.84^Aa^	66.46^Ab^	34.65^Ac^	13.58^Bd^				
AA (g/kg DM)	CK	2.09^Dab^	2.37^Ca^	1.71^Cbc^	0.90^Dd^	1.39^Bcd^	0.032	<0.001	<0.001	<0.001
	LP	2.49^Cb^	3.05^Ba^	2.32^Bb^	1.63^Cc^	3.06^Aa^				
	LX	3.52^Aa^	3.63^Aa^	2.56^Bc^	2.85^Abc^	3.31^Aab^				
	M	3.13^Bb^	3.67^Aa^	3.42^Aab^	2.41^Bc^	2.51^Ac^				
PA (g/kg DM)	CK	0.48^b^	0.58^a^	0.59^a^	ND	ND	0.005	<0.001	<0.001	<0.001
	LP	0.27	0.27	ND	ND	ND				
	LX	0.2	0.14	ND	ND	ND				
	M	ND	ND	ND	ND	0.59				
BA (g/kg DM)	CK	ND	ND	ND	ND	ND	0.013	<0.001	<0.001	<0.001
	LP	ND	3.26^Ba^	3.50^a^	ND	ND				
	LX	ND	ND	ND	ND	ND				
	M	ND	4.32^Aa^	3.52^b^	ND	ND				
NH_3_–N (g/kg TN)	CK	0.6^Ab^	0.6^Ab^	0.5^ABb^	0.9^Bb^	1.8^Ba^	0.02	<0.001	<0.001	<0.001
	LP	0.1^Bb^	0.2^Cb^	0.3^Bb^	0.2^Cb^	0.7^Ca^				
	LX	0.4^Ac^	0.4^Bc^	0.7^Abc^	1.1^Ab^	2.4^Aa^				
	M	0.1^Bc^	0.2^BCbc^	0.3^Bb^	0.2^Cbc^	0.7^Ca^				

*ND* not detected, *LA* lactic acid, *AA* acetic acid, *PA* propionic acid, *BA* butyric acid, *NH*_3_*–N* ammoniacal nitrogen, *TN* total nitrogen, *CK* control, *LP* FAE-producing *L. plantarum*, *LX* laccase and xylanase, *M* combination of FAE-producing *L. plantarum*, laccase and xylanase, *T* Treatment, *D* Aerobic exposure days, *T × D* Interacting Treatment and Days of Aerobic Exposure, *SEM* standard error of the mean

^A-D^Within a column and item, means without a common superscript differed (*P* < 0.05)

^a-e^Within a line, means without a common superscript differed (*P* < 0.05), the same letter indicates no significant difference (*P* >0.05)

### Impact of additives on the aerobic stability of mulberry

**Fig. 1:**
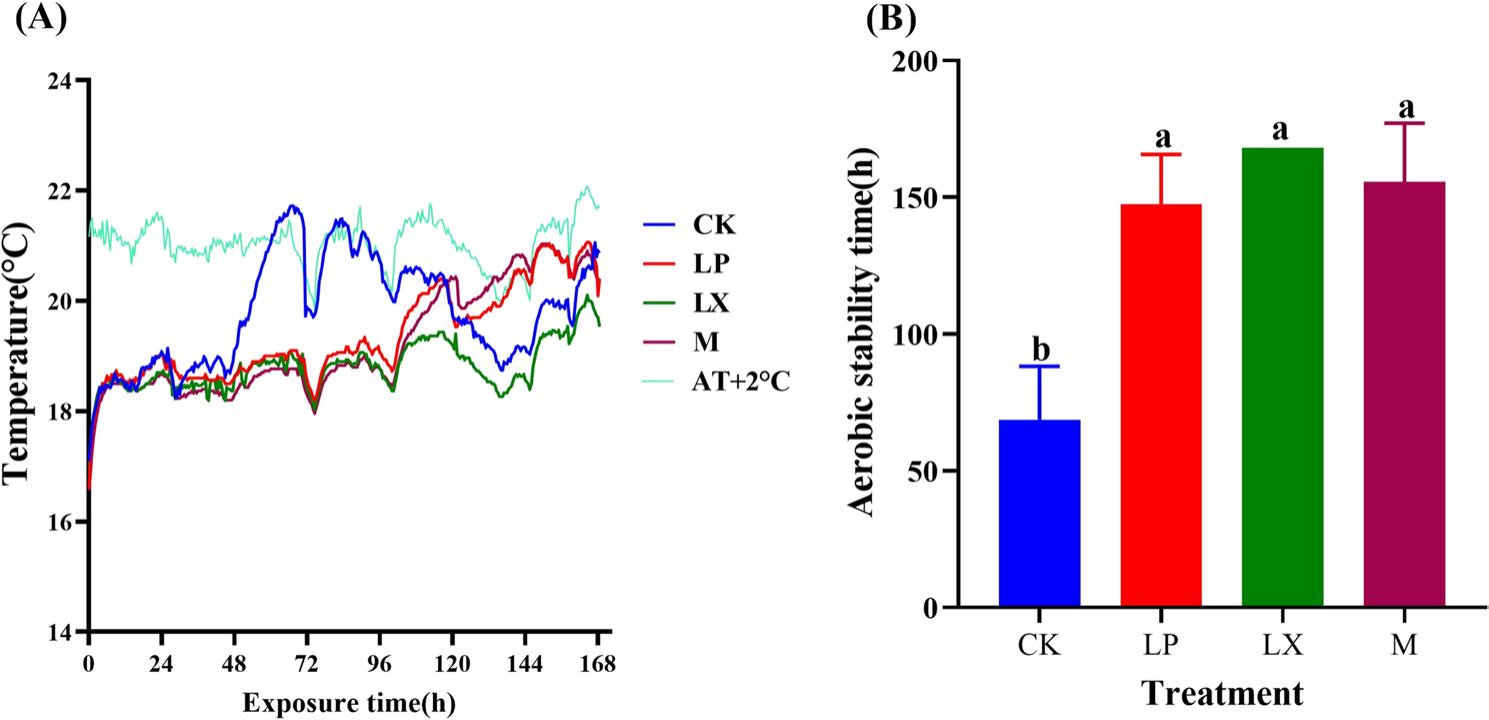
During aerobic exposure: **A** temperature change; **B** aerobic stabilization time; AT + 2 °C, Ambient temperature plus 2 °C; CK, control; LP, FAE-producing *L. plantarum*; LX, laccase and xylanase; M, combination of FAE-producing *L. plantarum*, laccase and xylanase; a-d indicate differences between treatments (*P* < 0.05); the same letter indicate no significant difference (*P* > 0.05)

According to the temperature pattern during aerobic exposure shown in Fig. 1A, the temperature of the CK group exceeded the ambient temperature by 2 °C from 69 to 71 h of aerobic exposure, which indicates that the forage began to deteriorate during this period. When the temperature increased to less than 2 °C, the silage was relatively stable and less prone to spoilage. During aerobic exposure, the LX treatment group did not exhibit obvious signs of aerobic spoilage. The LP treatment group first exceeded the ambient temperature by 2 °C from 142 to 146 h, whereas the M treatment group reached this threshold between 144 and 146 h, LP treatment was similar to M. This sustained temperature increase indicated the onset of mulberry forage decay. As shown in Fig. 1B, the aerobic stabilization time of the additive-treated groups was significantly longer than that of the CK group (*P* < 0.05), and the aerobic stability, in descending order, was as follows: LX, M, LP, and CK.

### Effect of additives on the chemical composition of mulberry silage

**Fig. 2:**
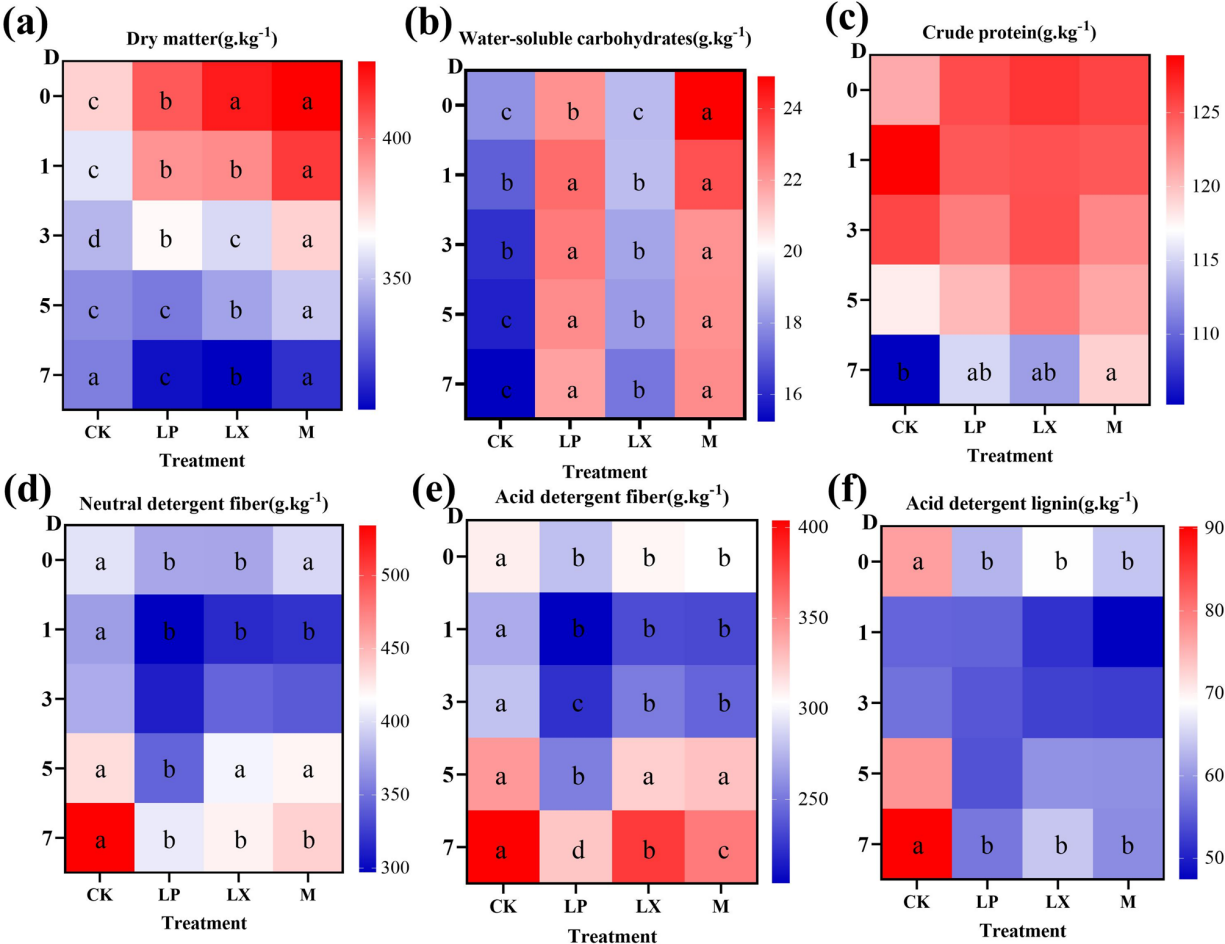
Changes in (**a**) dry matter; **b** water-soluble carbohydrates; **c** crude protein; **d** neutral detergent fiber; **e** acid detergent fiber; and (**f**) acid detergent lignin during aerobic exposure of mulberry silage. CK, control; LP, FAE-producing *L. plantarum*; LX, laccase and xylanase; M, combination of FAE-producing *L. plantarum*, laccase and xylanase. D, Aerobic exposure days; a-d indicate differences between treatments *P* < 0.05); the same letter indicate no significant difference (*P >* 0.05)

As shown in Fig. 2a, with increasing aerobic exposure time, the DM content of each treatment group gradually decreased. During the first 5 days of aerobic exposure, the DM content of the M treatment group was significantly greater than that of the other treatment groups (*P* < 0.05). This could be attributed to the synergistic effect among laccase, xylanase, and FAE-producing *L. plantarum*. As shown in Fig. 2b Compared with CK and M, LP treatment and M treatment significantly increased the WSC content during aerobic exposure of mulberry silage (*P* < 0.05). However, the LX treatment showed no significant difference from CK in the first 3 days of aerobic exposure (*P* > 0.05). Figure 2c indicates that the CP content gradually decreased as the aerobic exposure time increased. There was no significant difference among the treatments during the first 5 days of aerobic exposure, whereas on the 7th day, the CP content of the M treatment exhibited significantly greater effects than that of the control (*P* < 0.05).

The contents of NDF and ADF in the LP, LX and M treatments were significantly lower than those in the CK treatment (*P* < 0.05) (Fig. 2d, e, f), especially the LP treatment is the most effective. The additive treatments had significantly lower levels of ADL than controls on days 0 and 7 of aerobic exposure (*P* < 0.05), whereas there were no significant differences on days 1–5 of aerobic exposure (*P* > 0.05).

### Effect of additives on the lignocellulose of mulberry silage

**Fig. 3:**
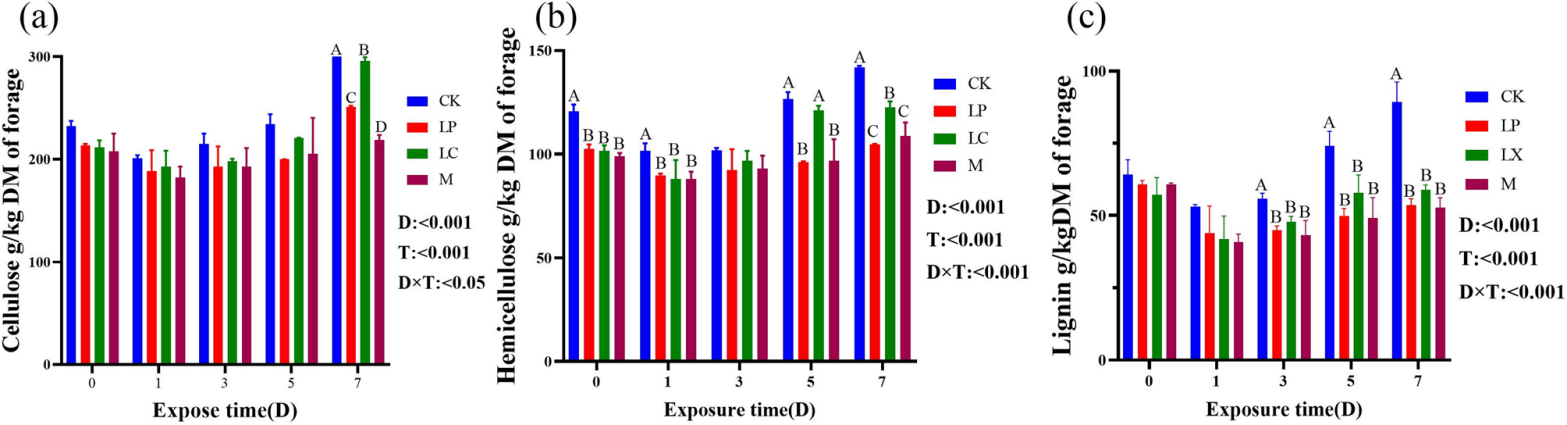
Changes in (**a**) cellulose, (**b**) hemicellulose, and (**c**) lignin content of silage mulberry during the aerobic period under different treatments. CK, control; LP, FAE-producing *L. plantarum*; LX, laccase and xylanase; M, combination of FAE-producing *L. plantarum*, laccase and xylanase; T, Treatment; D, Aerobic exposure days, T × D, Interacting Treatment and Days of Aerobic Exposure; A-D, indicate differences between treatments (*P* < 0.05); the same letter indicate no significant difference (*P* > 0.05)

There was no significant difference in the cellulose content of mulberry silage during the first 5 days of aerobic exposure, but the additive treatment was significantly lower than CK on the 7th day of aerobic exposure (*P* < 0.05), especially the M treatment had the lowest content (Fig. 3a). During aerobic exposure, hemicellulose content of additive treatments was significantly lower than that of CK (*P* < 0.05). As shown in Fig. 3c, and lignin content of additive treatment group was significantly lower than that of control group on days 3–7 of aerobic exposure (*P* < 0.05), and there was no significant difference between additive treatments (*P* > 0.05).

### Changes in the activities of three types of proteases during aerobic exposure of mulberry silage

**Fig. 4:**
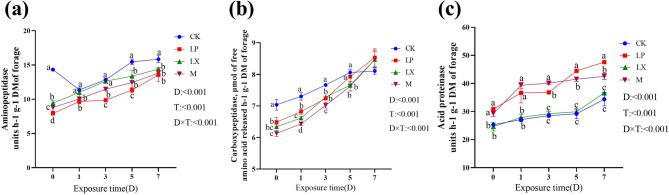
Changes in the activities of (a) aminopeptidase, (b) carboxypeptidases, and (c) acidic protein hydrolase during aerobic exposure of mulberry silage. CK, control; LP, FAE-producing *L. plantarum*; LX, laccase and xylanase; M, combination of FAE-producing *L. plantarum*, laccase and xylanase; T, Treatment; D, Aerobic exposure days, T × D, Interacting Treatment and Days of Aerobic Exposure; a-d, indicate differences between treatments (*P* < 0.05); the same letter indicate no significant difference (*P* > 0.05)

As depicted in Fig. 4a, b, c, with increasing aerobic exposure time, the activities of the three proteases increased. During aerobic exposure, the aminopeptidase activities of FAE-producing *L. plantarum* (LP and M) treatments were significantly lower than those of CK and LX treatments (*P* < 0.05), and the LX treatment was not significantly different from the control on days 1 and 3(*P* > 0.05). Carboxypeptidase activities of all additive treatments were significantly lower than those of the CK treatment during the first 5 days of aerobic exposure (*P* < 0.05) and were not significantly different on day 7. Acidic protein hydrolase activity was significantly higher in LP and M treatments than in CK and LX (*P* < 0.05), with no significant difference between CK and LX treatments (*P* > 0.05).

### In *vitro *digestibility analysis of mulberry fermentation for 60 days

**Fig. 5:**
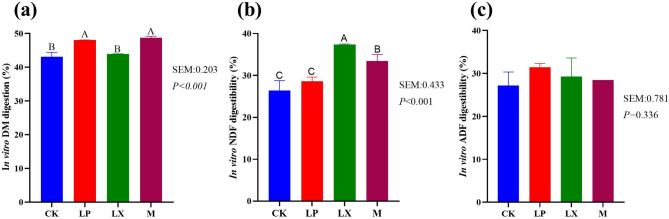
In vitro digestibility of (a) DM, (b) NDF and (c) ADF of mulberry silage for 60 days. IVDMD, in - vitro dry matter digestibility; IVNDFD, in - vitro neutral detergent fiber digestibility; IVADFD, in - vitro acid detergent fiber digestibility; CK, control; LP, FAE-producing *L. plantarum*; LX, laccase and xylanase; M, combination of FAE-producing *L. plantarum*, laccase and xylanase; A-D, indicate differences between treatments (*P* < 0.05); the same letter indicate no significant difference (*P* > 0.05)

Figure 5a shows that FAE-producing *L. plantarum* (LP and M) treatments increased IVDMD (*P* < 0.05), with no significant difference between LX and CK (*P* > 0.05), and no significant difference between LP and M (*P* > 0.05). Figure 5b IVNDFD was significantly higher in the LX treatment than in M, which was significantly higher than in the CK and LP treatments (*P* < 0.05). IVNDFD was no significant difference between CK and LP treatments (*P* > 0.05). Figure 5b shows that additive treatments had no significant effect on IVADFD (*P* > 0.05).

### Changes in microbial communities during aerobic exposure of mulberry silage

As shown in Table 3, as the duration of aerobic exposure increased, the LAB population gradually increased. Conversely, yeast and *Coliform* bacteria started to grow from an initial count of zero. Compared with those in the LX and control groups, significantly lower levels were detected in the LP and M treatments (*P* < 0.05). In both the LP and M groups, the LAB population tended to increase with increasing pH. At the commencement of aerobic exposure (day 0), no growth of *Coliform* bacteria or yeast was detected in any of the treatments. In the CK treatment, the growth of *Coliform bacteria* and yeast commenced on day 1 of aerobic exposure. The additive treatment significantly reduced the number of yeasts (*P* < 0.05).

**Table 3:** Changes in microbial counts during aerobic exposure of silage mulberry

Parameter	Treatment (T)	Days of aerobic exposure (D)					SEM	*P-value*		
		Day 0	Day 1	Day 3	Day 5	Day 7		T	D	T × D
LAB（log_10_ CFU/gFM）	CK	5.53^Bc^	5.6^4c^	6.19^Ab^	6.48^Ab^	7.41^Aa^	0.021	<0.001	<0.001	<0.001
	LP	4.15^Cd^	4.98^Cc^	4.92^Bc^	6.15^Bb^	6.93^Ba^				
	LX	6.09^A^	6.20^A^	6.07^A^	5.47^C^	5.63^C^				
	M	4.00^De^	4.39^Dd^	5.04^Bc^	6.28^ABb^	6.98^Ba^				
*Coliform *bacteria(log_10_ CFU/gFM）	CK	ND	5.72^Ad^	6.07^Ac^	6.32^Ab^	7.06^Aa^	0.013	<0.001	<0.001	<0.001
	LP	ND	5.11^Bd^	4.74^Bc^	6.20^Ab^	6.84^Ba^				
	LX	ND	ND	ND	4.15^Bb^	5.00^Ca^				
	M	ND	ND	4.73^Bc^	6.16^Ab^	6.99^ABa^				
Yeast (log_10_ CFU/g FM）	CK	ND	6.03^Ac^	6.07^Ac^	6.46^Ab^	6.91^Aa^	0.011	<0.001	<0.001	<0.001
	LP	ND	ND	ND	6.11^Bb^	6.76^Ba^				
	LX	ND	4.83^B^	ND	ND	ND				
	M	ND	ND	4.24^Bc^	6.18^Bb^	ND				

*ND* not detected, *LAB* lactic acid bacteria, *CFU* colony-forming units, *CK* control, *LP* FAE-producing *L. plantarum*, *LX* laccase and xylanase, *M* combination of FAE-producing *L. plantarum*, laccase and xylanase, *T* Treatment, *D* Aerobic exposure days, *T × D* Interacting Treatment and Days of Aerobic Exposure, *SEM* standard error of the mean

^A-D^Within a column and item, means without a common superscript differed (*P* < 0.05)

^a-e^Within a line, means without a common superscript differed (*P* < 0.05), the same letter indicate no significant difference (*P* >0.05)

High-throughput sequencing of 16 S rRNA gene amplicons was employed to comprehensively elucidate the bacterial communities present within the raw materials and silages. The alpha diversity metrics, encompassing observed species, Good’s coverage, abundance-based coverage estimator (ACE), Chao1, and Shannon indices, were calculated for fresh samples, as well as for silage samples subjected to aerobic exposure for 0, 3, and 7 days; these results are presented in Table 4. Notably, the Good’s coverage values for all samples exceeded 0.98, signifying that the sequencing depth was adequate to accurately reflect the true composition of the samples. The Chao1 and ACE indices of the M treatment were significantly higher than those of other treatments (*P* < 0.05). The Shannon indices after aerobic exposure of silage were higher than those of fresh samples.

**Table 4:** Diversity and richness of bacterial flora in mulberry silage

Days	Sample name		Observed species	chao1	Shannon	ACE	Good's coverage
	FM		47.33B	429.40B	0.18C	93.75B	0.998
0d	CK0		103.67B	142.80B	3.10A	160.94B	0.996
	LP0		81.33B	113.71B	1.34BC	132.61B	0.997
	LX0		84.33B	124.99B	2.35AB	130.07B	0.997
	M0		60.33B	86.40B	1.40BC	92.97B	0.999
3d	CK3		80.00B	116.98B	2.51AB	129.11B	0.997
	LP3		116.67B	172.04B	1.45BC	183.74B	0.995
	LX3		34.00B	42.92B	1.88AB	42.92B	0.999
	M3		126.33B	176.06B	2.53AB	189.65B	0.995
7d	CK7		105.33B	152.46B	2.04AB	177.27B	0.996
	LP7		73.33B	206.37B	1.24BC	168.00B	0.996
	LX7		42.67B	50.97B	2.01AB	58.46B	0.999
	M7		310.67A	429.40A	2.29AB	443.20A	0.988
		SEM	11.664	17.1900	0.1140	16.221	0.0001
	*P-value*		0.014	0.024	0.0050	0.012	0.005

*Abbreviations*: *ACE* abundance-based coverage estimator, *CK* control group, *LP* L. plantarum, *LP* FAE-producing *L. plantarum*, *LX* laccase and xylanase, *M* combination of FAE-producing *L. plantarum*, laccase and xylanase

Different letters indicate significant differences in the same column (*P* < 0.05); the same letter indicate no significant difference (*P *>0.05)

**Fig. 6:**
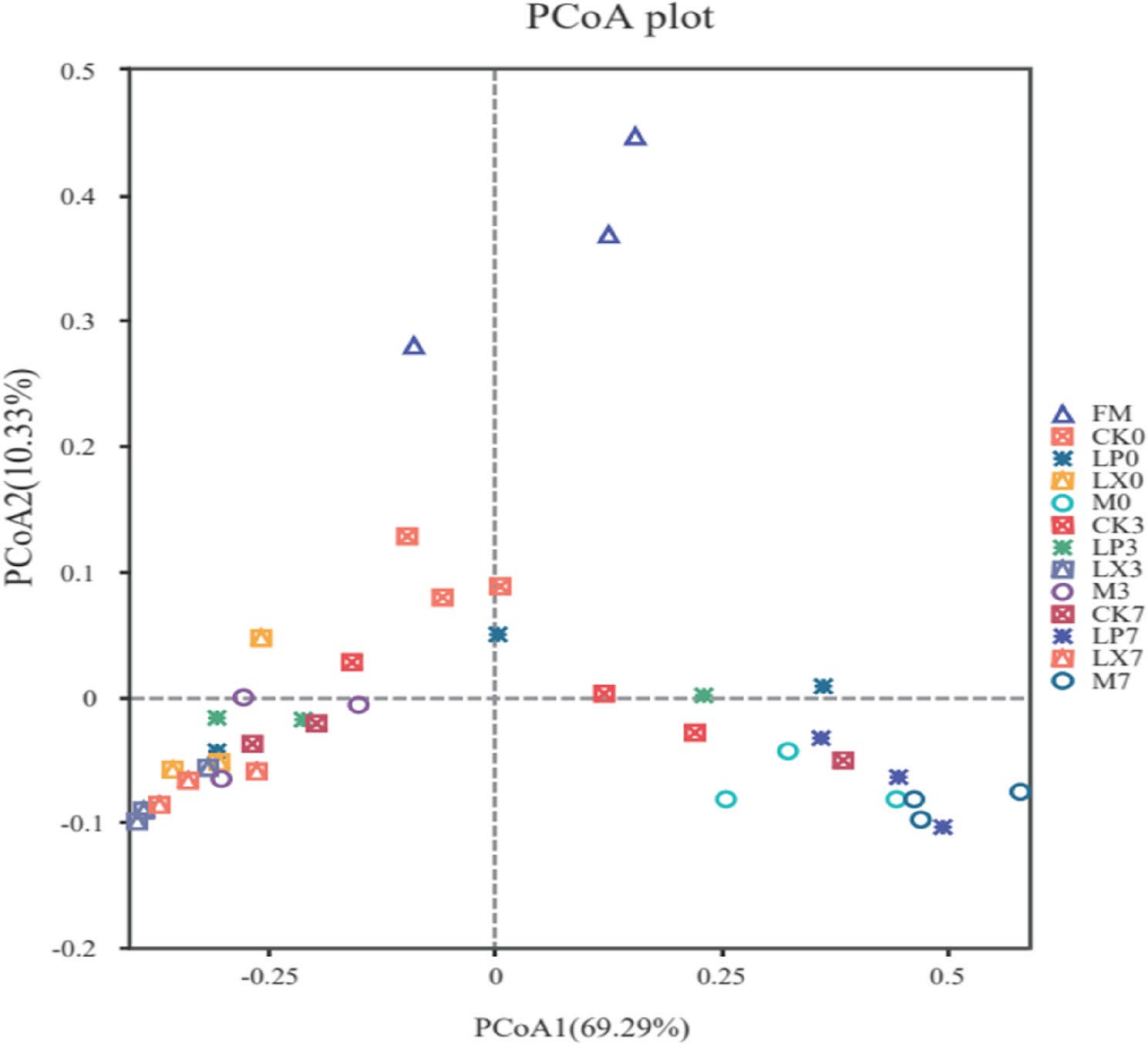
Principal coordinate analysis (PCoA) based on 0, 3 and 7 days of mulberry silage aerobic exposure. FM, fresh mulberry; CK, control; LP, FAE-producing *L. plantarum*; LX, laccase and xylanase; M, combination of FAE-producing *L. plantarum*, laccase and xylanase

As presented in Fig. 6, Component 1 and Component 2 explained 69.29% and 10.33% of the total variance, respectively. After 60 days of ensiling, on the third day of aerobic exposure, the LP, LX, and M feed samples were clearly differentiated from CK. By the seventh day of aerobic exposure, LP and M were distinctly separated from LX and CK. This result demonstrated significant disparities in the microbial communities between inoculated and no inoculated silage.

**Fig. 7:**
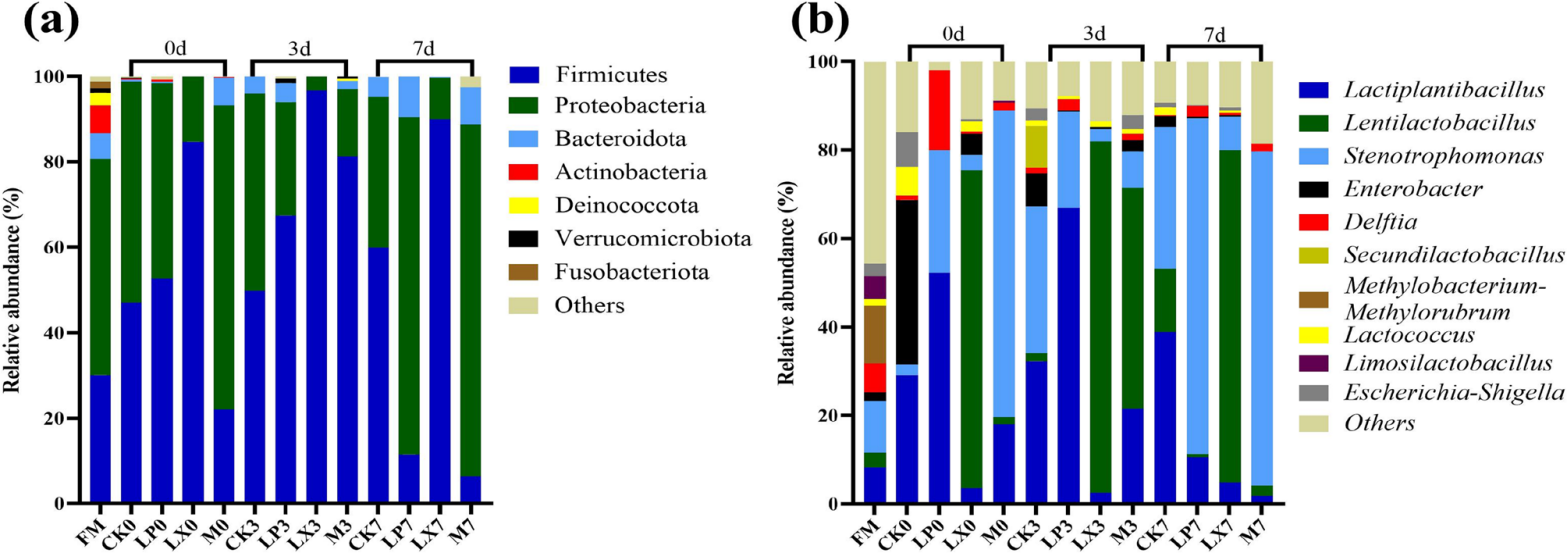
The relative abundance of the bacterial community in mulberry silage on days 0, 3, and 7 of aerobic exposure, at (**a**) the phylum level and (**b**) the genus level. based FM, fresh mulberry; CK, control; LP, FAE-producing *L. plantarum*; LX, laccase and xylanase; M, combination of FAE-producing *L. plantarum*, laccase and xylanase

The microbial community structure depicted in Fig. 7a indicates that, following ensiling, in aerobically exposed whole-plant mulberry silage, the bacterial community was dominated by the phyla *Firmicutes* and *Proteobacteria*. The LX treatment significantly increased the relative abundance of *Firmicutes*. Conversely, the M treatment led to a notable increase in the relative abundance of *Proteobacteria* on both day 0 and day 7. As shown in Fig. 7b, the bacterial composition of mulberry silage is predominantly characterized by the genera *Lactiplantibacillus*, *Lentilactobacillus*, and *Stenotrophomonas*. The introduction of additives led to a reduction in the richness of bacterial species in mulberry silage. During the aerobic exposure process of silage, the dominant microbial community in the LX treatment group was *Lentilactobacillus*. LP treatment resulted increase in the relative abundance of *Lactiplantibacillus* during the first 3 days of aerobic exposure and a decrease on day 7.

**Fig. 8:**
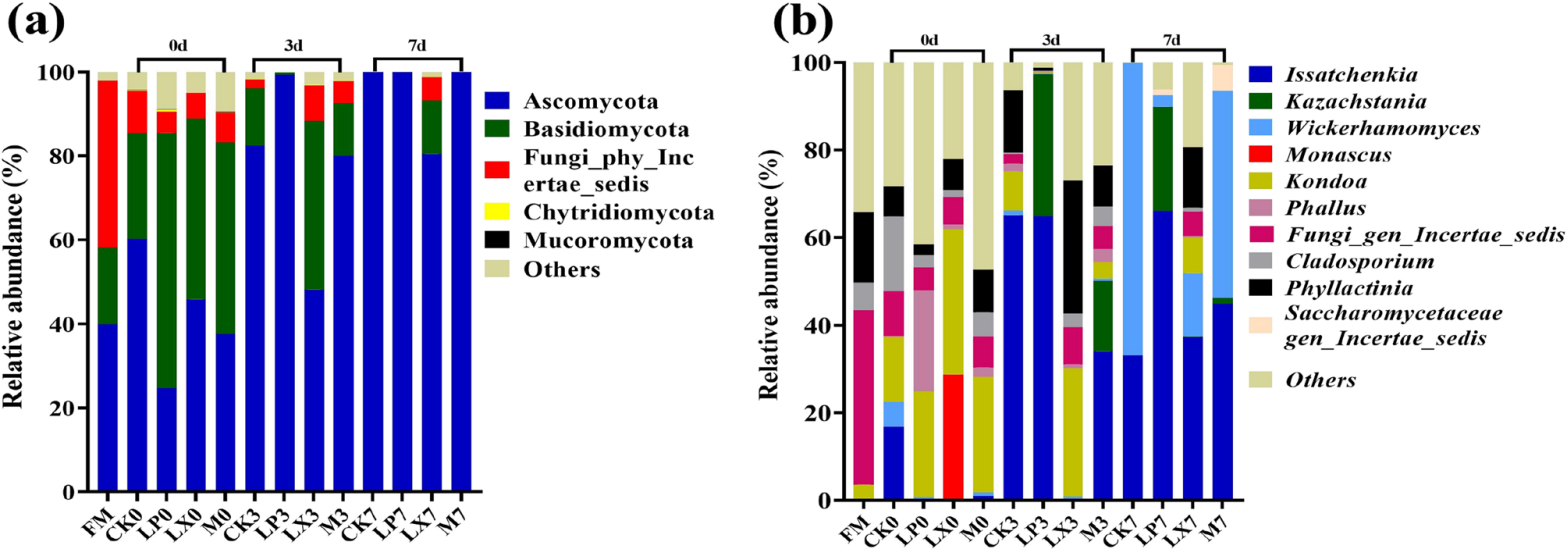
The relative abundance of the fungi community in mulberry silage on days 0, 3, and 7 of aerobic exposure, at (a) the phylum level and (b) the genus level. based FM, fresh mulberry; CK, control; LP, FAE-producing *L. plantarum*; LX, laccase and xylanase; M, combination of FAE-producing *L. plantarum*, laccase and xylanase

As depicted in Fig. 8, *Ascomycetes* was the dominant phylum throughout the aerobic phase of mulberry silage fermentation. The LX treatment increased the relative abundance of *Basidiomycota* during the aerobic exposure of mulberry silage. The relative abundance of *Issatchenkia* in the LX treatment significantly increased on day 7, while *Issatchenkia* was barely detectable on days 0 and 3. Treatment with FAE - producing *L. plantarum* (LP and M) increased the relative abundance of *Kazachstania* on days 3 and 7 of aerobic exposure. The additive treatments increased the relative abundance of *Phyllactinia*, especially in the LX treatment group on days 3 and 7 of aerobic exposure. The relative abundance of *Wickerhamomyces* increased on day 7 of aerobic exposure. However, compared with CK, all additive treatments reduced the relative abundance of *Wickerhamomyces*, especially the LP and LX treatments.

**Fig. 9:**
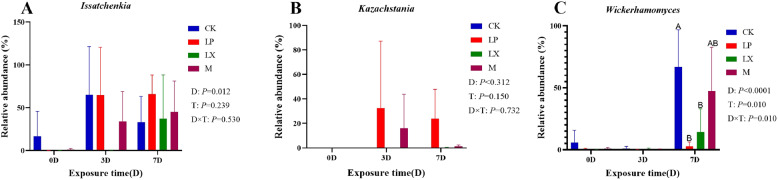
Changes in the relative abundances of dominant fungi genera: (A) *Issatchenkia*; (B) *Kazachstania*; (C) *Wickerhamomyces*. CK, control; LP, FAE-producing *L. plantarum*; LX, laccase and xylanase; M, combination of FAE-producing *L. plantarum*, laccase and xylanase. Means within the different treatments (A, B, C) with different letters differ significantly from each other (*P* < 0.05), the same letter indicates no significant difference (*P* > 0.05)

**Fig. 10:**
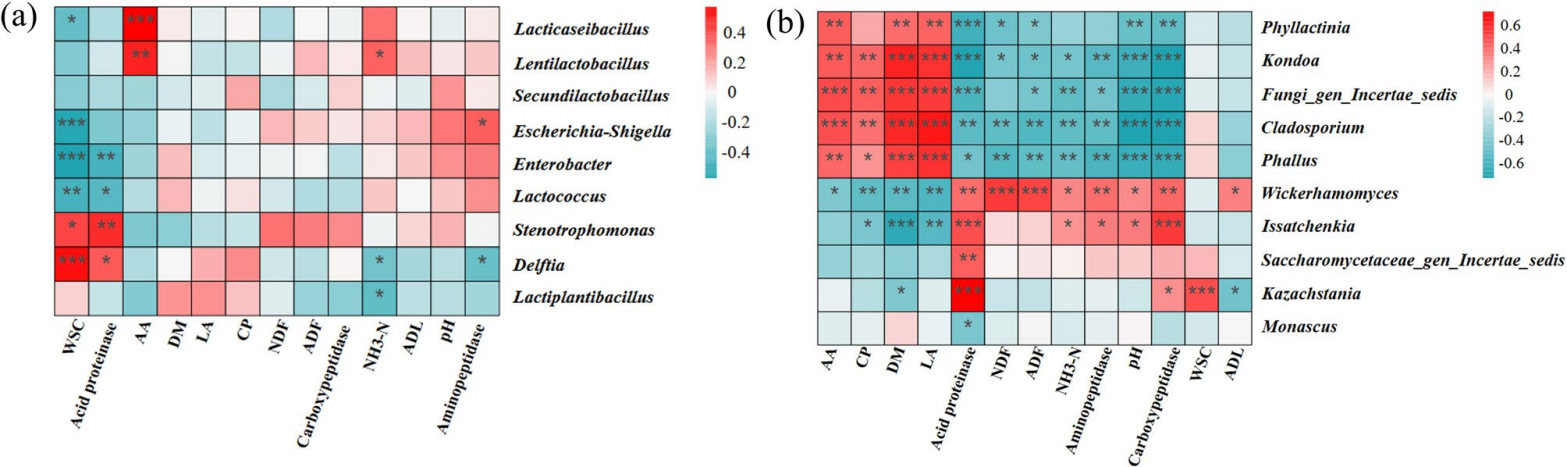
Spearman analysis between silage parameters and bacterial genus (**a**) fungal genus (**b**) during aerobic exposure.LA, lactic acid; AA, acetic acid; DM, dry matter; WSC, water-soluble carbohydrates; NDF, neutral detergent fiber; ADF, acid detergent fiber; ADL, acid detergent lignin; CP, crude protein; NH_3_-N, ammonia nitrogen.*Significance at *P < 0.05*; **significance at *P* < 0.01; ***significance at *P* *< 0.001*

As depicted in Fig. 9, on day 7, the relative abundance of *Wickerhamomyces* in the CK treatment was significantly higher than that in the LX and LP treatments (*P* < 0.05). In the LP treatment, the relative abundance of *Kazachstania* began to increase after day 3, but due to a large margin of error, the difference was not significant (*P* > 0.05) As depicted in Fig. 10, the NH₃–N content of mulberry silage was negatively correlated with the relative abundance of *Lactiplantibacillus* within the bacterial community (*P* < 0.05). The AA content was positively correlated with the relative abundances of *Lacticaseibacillus*, *Lentilactobacillus*,* Phallus*, *Cladosporium*, *Phyllactinia* and *Kondoa* (*P* < 0.01) but negatively correlated with the relative abundance of *Wickerhamomyces* (*P* < 0.05). *Issatchenkia* and *Wickerhamomyces* were negatively correlated with LA (*P <* 0.01). *Phallus*, *Cladosporium*, *Phyllactinia* and *Kondoa* were positively correlated with LA (*P* < 0.01).

## Discussion

Compared with corn and oat, which are typical conventional forages, mulberry exhibits higher levels of CP, NDF, ADF, and ADL [31, 32]. The high fiber content can restrict the efficiency of mulberry utilization; consequently, fiber degradation is crucial for enhancing the utilization of mulberry silage. Hemicellulose is a part of NDF, hemicellulose hydrolysis is the main route for reducing NDF, as hemicellulose degrades, the bond between lignin and cellulose weakens, causing lignin to partially dissolve with hemicellulose and reducing the ADF content [33]. In the LP treatment, the NDF and ADF contents were significantly lower, likely because the FAE-producing *L. plantarum* degraded hemicellulose [34], leading to a significantly reduced hemicellulose content in the LP treatment. *L. plantarum* produces LA, which reduces the pH and thereby creates an acidic environment. This is likely due to the acid hydrolysis of more digestible cell wall fractions during the ensiling process, thus decreasing the contents of NDF and ADF [8]. At the later stage of aerobic exposure, the ADL content in the additive treatment was significantly lower than that in the CK group, which can be attributed to the certain delignification ability of the FAE-producing *L. plantarum* [35]. Cellulase and xylanase hydrolyze plant cell walls to generate fermentable sugars, thereby decreasing the NDF and ADF content in oat silage [14]. In this study, the combined treatment of laccase and xylanase had a similar effect. Xylanase breaks the chemical bond between lignin and carbohydrates, feruloyl esterase can cleave the ester bond between lignin and hemicellulose, and laccase degrades lignin. Xylanases hydrolyze hemicelluloses, thereby improving the accessibility of cellulases to cellulose [36]. When lignin is degraded, the strong bonds among lignin, cellulose, and hemicellulose are disrupted; as a result, the utilization of cellulose and hemicellulose is enhanced [37]. We hypothesized that during the ensiling process, the FAE-producing *L. plantarum* degraded lignocellulose [10]. The additives were more effective in fiber degradation during the later stages of aerobic exposure, thereby enhancing the effectiveness of mulberry silage during the later stages of aerobic exposure. Laccase and xylanase increase the IVNDFD, while *L. plantarum* increase the IVDMFD. No substantial variation was detected in the IVADFD. Research has indicated that the molecular structure of carbohydrates can affect the rumen degradation rate of NDF in alfalfa and concentrate-based feed [38]. The disparities in IVNDFD between the control and additive treatments could be partly attributed to variations in NDF degradation rates [39]. When compared with CK treatment, translation LP had no significant effect on IVNDFD. The LX treatment exhibited a significantly more pronounced effect in augmenting the IVNDFD. This observed outcome is plausibly ascribable to the direct enzymatic degradation of lignin by laccase. Such degradation effectively disrupts the lignin-hemicellulose defensive matrix, thereby enhancing the accessibility of digestive enzymes to the substrate [5] An in-depth study revealed that FAE-producing bacteria play a key role in lowering the pH of silage. This finding was consistent with the results of previous research, indicating that these bacteria could effectively promote LA production and acidification in silage, thus contributing to improving the preservation and quality of silage [40]. Aerobic spoilage of silage could be considered to have occurred when the pH of the silage increased by 0.5 from the initial value [41]. According to pH the mulberry silage CK started to spoilage on aerobic day 5, LP and M started to spoilage on day 7, whereas LX remained essentially unaltered during aerobic exposure. The LA content significantly decreased after 1 day of aerobic exposure, which was attributed to the breakdown and utilization of LA by microorganisms such as yeasts and aerobes [42]. In the late stage of aerobic exposure, the reduction of utilizable substances leads to the utilization of LA by microorganisms, resulting in a decrease in LA content. On day 7 of the M treatment, the abundance of *Wickerhamomyces* increased more than that in the LP treatment. This fungus is negatively correlated with LA, which may explain the sharp decrease in LA content in the M treatment on day 7. The LA content in the group treated with M was more than 20 times that of AA, yet the oxygen stability was much higher. It may be that the increase in LA content causes the pH of silage to decrease. By rapidly reducing the pH to below 4.2, LA significantly inhibits the growth of yeasts and molds, extending the aerobic stability period of silage to more than 72 h [43]. Additive treatments increased the AA content of mulberry silage during aerobic exposure. In silage fermentation, increasing the AA content could increase aerobic stability and maintain storage quality, inhibiting the growth of yeasts and undesirable bacteria and helping LAB become the dominant flora, thereby improving silage fermentation quality [44]. This is one of the reasons why the additive treatment improves aerobic stability. Aerobic microorganisms (yeast and aerobic bacteria) oxidize organic acids and WSCs to produce carbon dioxide and water, generating heat during growth and reproduction, which leads to an increase in temperature and results in a decrease in aerobic stability [45, 46]. This was most likely because a large amount of AA was produced, which inhibited the growth of moulds and yeasts during aerobic exposure [47]. This is because the addition of FAE-producing LABs and degrading enzymes may lead to the generation and retention of more WSCs via the decomposition of lignocellulose and rapid and dominant lactic acid fermentation, which can inhibit the growth of undesirable bacteria during the aerobic stage [48]. The WSC content of the LX treatments was not significantly different from that of the CK. This may be because the pH value of the silages did not decrease below 4.2, resulting in the continued utilization of sugar by other harmful microorganisms [49] Carboxypeptidases, aminopeptidases and acid proteases are the main plant enzymes involved in protein degradation. These enzymes exhibit different behaviors under different pH and temperature conditions and have different sensitivities to inhibitors. The overall extent of protein hydrolysis in silage is largely dependent on pH, which has a strong influence on the activity and stability of proteases [9, 29]. This suggests that the addition of FAE-producing *L. plantarum* facilitates the inhibition of protein degradation, thereby reducing the ammoniacal nitrogen content. This may be because the additive accelerates *Lactobacillus* fermentation and inhibits the activity of spoilage bacteria and the cellular respiration in harvested plant tissues [34]. The higher ammoniacal nitrogen content of the LX treatments was probably due to the higher pH resulting in elevated protease activity and protein degradation. The increase in acid protease activity, in conjunction with an elevated abundance of peptides and free amino acids, strongly suggests the pivotal role of acid proteases in protein hydrolysis. Treatments involving FAE-producing *L. plantarum* (LP and M) increase acid protease activity by modulating the pH value This high coverage ensures reliable and in-depth analysis of the microbial community structure, facilitating a more precise understanding of the microbial dynamics within the studied materials [50]. During aerobic exposure, the number of *Lactiplantibacillus* decreased, which may be due to the proliferation of *Enterobacter* during silage fermentation, which converted LA produced by *Lactiplantibacillus* to AA. Since AA is less acidic than LA, the increase in AA content will increase the pH value of the silage and disrupt the growth environment of *Lactiplantibacillus* [51]. The introduction of additives led to a reduction in the richness of bacterial species in mulberry silage. *Lentilactobacillus* was identified as the predominant microbial genus in the LX treatment group. Extensive research evidence has shown that inoculation with *Lentilactobacillus buchneri* significantly elevates the aerobic stability of maize silage. This bacterium’s antioxidant characteristics play a crucial role in enhancing the aerobic stability of mulberry silage [52]. *Kazakhstani unispora* was determined to be the principal yeast species present in aerobically deteriorated maize silage and was among the species documented in the study [53]. Once the silage is exposed to air, aerobic microorganisms use LA as a growth substrate, leading to an increase in the pH of the silage [43] A high-pH environment favors the growth of filamentous fungi, and the proliferation of filamentous fungi, in turn, causes an increase in silage temperature. This phenomenon explains why the aerobic stability of the additive-treated group was greater than that of the CK group. *Lactobacillus*, *Stenotrophomonas* was positively correlated with AA and has the ability to convert some of the LA to AA under acidic conditions as documented by Muck et al. [54]. *Lentilactobacillus* increased AA content and thus improved aerobic stability. *Stenotrophomonas* was positively correlated with acidic proteases, *Stenotrophomonas* spp. have low nutrient requirements but are considered unfavorable for silage due to their ability to degrade proteins [55].The relative abundance of *Lactiplantibacillus*, *Lentilactobacillus*, *Phyllactinia*, *Cladosporium* and *Kondoa* was positively correlated (*P* < 0.05) with AA levels, which may be due to the fact that these taxa have different metabolic functions. In contrast, the relative abundance of *Wickerhamomyces* was negatively correlated (*P* < 0.05) with AA levels. These findings suggest that these microorganisms may be involved in the aerobic stabilization of mulberry silage Overall, the application of additives significantly enhanced the fermentation quality of mulberry silage. The M treatment exhibited a pronounced positive impact on in vitro fermentation. Moreover, these treatments effectively improved the aerobic stability of mulberry silage, with the LX treatment affording the highest degree of stability. The utilization of mulberry as feed not only broadens the spectrum of available feed resources but also enhances feeding efficiency.

## Conclusion

This study revealed that the addition of FAE-producing *L. plantarum*, laccase and xylanase increased the LA and AA contents and decreased the pH of mulberry silage, reducing the proliferation of harmful bacterial strains. The aerobic stability of the LX treatment group was the greatest, and that of the M group was similar to that of the control group. Moreover, the additive treatments reduced the species richness of mulberry silage. *Lentilactobacillus* became the dominant bacterial genus in the LX treatment group, which might have contributed to the increase in the aerobic stability of the mulberry silage. Overall, the addition of laccase, xylanase and FAE *L. plantarum* synergistically improved the fermentation and aerobic stability of mulberry silage

## Data Availability

The raw sequence data have been deposited in the sequence read archive at the NCBI (https://www.ncbi.nlm.nih.gov/) under accession number PRJNA1244789.
